# Application of the Flexible CO2 Laser in Minimally Invasive Laminectomies: Technical Note

**DOI:** 10.7759/cureus.628

**Published:** 2016-06-02

**Authors:** Namath S Hussain, Mick Perez-Cruet

**Affiliations:** 1 Department of Neurosurgery, Penn State Hershey Medical Center; 2 Michigan Head and Spine Institute, Oakland University William Beaumont School of Medicine

**Keywords:** co2 laser, spinal stenosis, laminectomy, minimally-invasive, spine surgery

## Abstract

Background

Minimally invasive laminectomy is a very effective surgical method for treating lumbar stenosis. However, this technique can be technically difficult, especially in patients suffering from severe stenosis. The contralateral decompression from a unilateral approach can result in durotomy during removal of the hypertrophied ligamentum flavum. This complication can be difficult to treat through a small working channel.

Objective

To detail our group’s operative experience with the CO_2_ laser and discuss our results and previous studies in the literature reporting results.

Methods

The CO_2_ laser (Omniguide, Boston, MA) was investigated in the surgical ablation of the contralateral ligamentum flavum during minimally invasive laminectomies. Forty levels have been investigated thus far. The amount of voltage needed to adequately desiccate and remove the ligamentum flavum safely as well as the effectiveness of this technique were investigated.

Results

The contralateral ligamentum flavum could be removed effectively using the 9 to 11 watt continuous wavelength (10,600 nanometer) power setting on the CO_2_ laser. Shrinkage of the contralateral ligamentum flavum facilitated its removal using a number 2 Kerrison Punch. No durotomies occurred, and the use of the laser did not significantly lengthen operative times.

Conclusions

The CO_2_ laser appears to be a useful tool in the armamentarium of instruments available to the minimally invasive spine surgeon and may help to reduce the incidence of durotomies when performing minimally invasive laminectomies.

## Introduction

Minimally invasive spine surgery has recently experienced a renaissance due to improved technology and investment in instrumentation designed specifically to be used through tubular dilators. One specific potential downside to the use of the minimally invasive method is the decreased ability to access certain types of pathology due to difficulty looking around corners. This problem can be alleviated through the use of an endoscope, but many spine surgeons are not very comfortable using endoscopes, and this may lead to increased operating rooms times and the concomitant complications that may result.

Collaborations between surgeons and industry have provided new products for better visualization to alleviate these problems. The CO_2_ laser is an older technology originally developed in 1964 that is being applied in a new fashion due to advances in flexible fiber technology and optics developed in 2008 to allow for a flexible laser delivery device [1]. We have found the laser to be useful during spinal decompression procedures for lumbar spinal stenosis when accessing and removing the contralateral ligamentum flavum. This obviates the need for dural or nerve root retraction. This bone and ligament removal is traditionally performed with sharp curettes and biting rongeurs, which may tear the dura and cause a leak of spinal fluid. The laser provides a method of desiccating and removing the ligament without damaging the dura, spinal cord, or nerve roots through retraction or excessive manipulation. The laser is useful for minimally-invasive procedures specifically due to the small working space and narrow working channel afforded. This paper describes the technique using the laser during minimally-invasive laminectomies with photographs and details of operative nuances with illustrative cases.

The indications for the use of the CO_2_ laser in minimally invasive laminectomies include stenosis with any degree of ligamentum flavum hypertrophy requiring removal. The procedure is especially useful in cases of very severe stenosis where undercutting the lamina on the contralateral side during a minimally-invasive procedure can be facilitated by removing some ligament to provide more working room for drilling or cutting of the lamina. Relative contraindications include prior surgery where the ligament at that level has already been removed, or where scar tissue may make tissue planes difficult to discern. 

## Technical report

Using fluoroscopy to fashion the appropriate 3 cm midline incision, a 15 blade can be used to cut the skin, followed by monopolar electrocautery to incise the fascia and underlying soft tissue. Dissection down to the facet complex is undertaken. The k-wire and dilator tubes can then be docked onto the facet complex in a serial fashion. The largest dilator to be used can then be docked to the articular arm which is mounted to the table.

**Figure 1:**
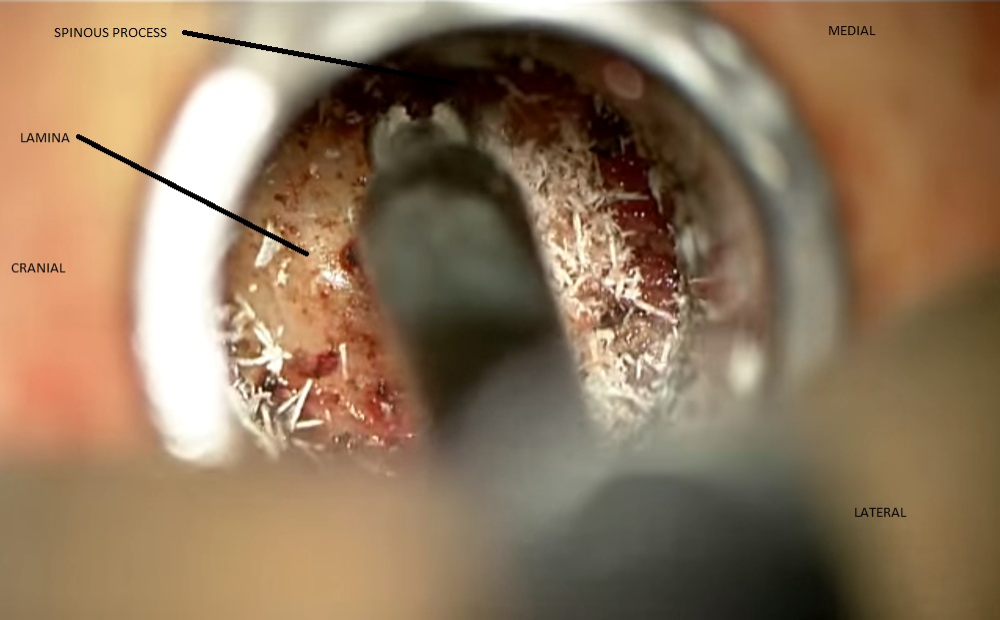
Minimally invasive laminectomy from a unilateral approach showing contralateral bony decompression

The facet complex and lamina can then be drilled down. A number 2 Kerrison rongeur can be used to remove the thinned-out lamina in a piecemeal fashion, taking care to avoid any under pressure on the underlying ligament or thecal sac. The ipsilateral ligament can be desiccated with bipolar electrocautery and removed with a combination of curettes and the Kerrison rongeur. The task then turns to the contralateral undecompressed aspect of the canal. The goal here is to undercut the contralateral lamina and the midline spinous process so as to preserve the spinous process and its overlying dorsal muscular attachments as seen in Figure 1.

**Figure 2:**
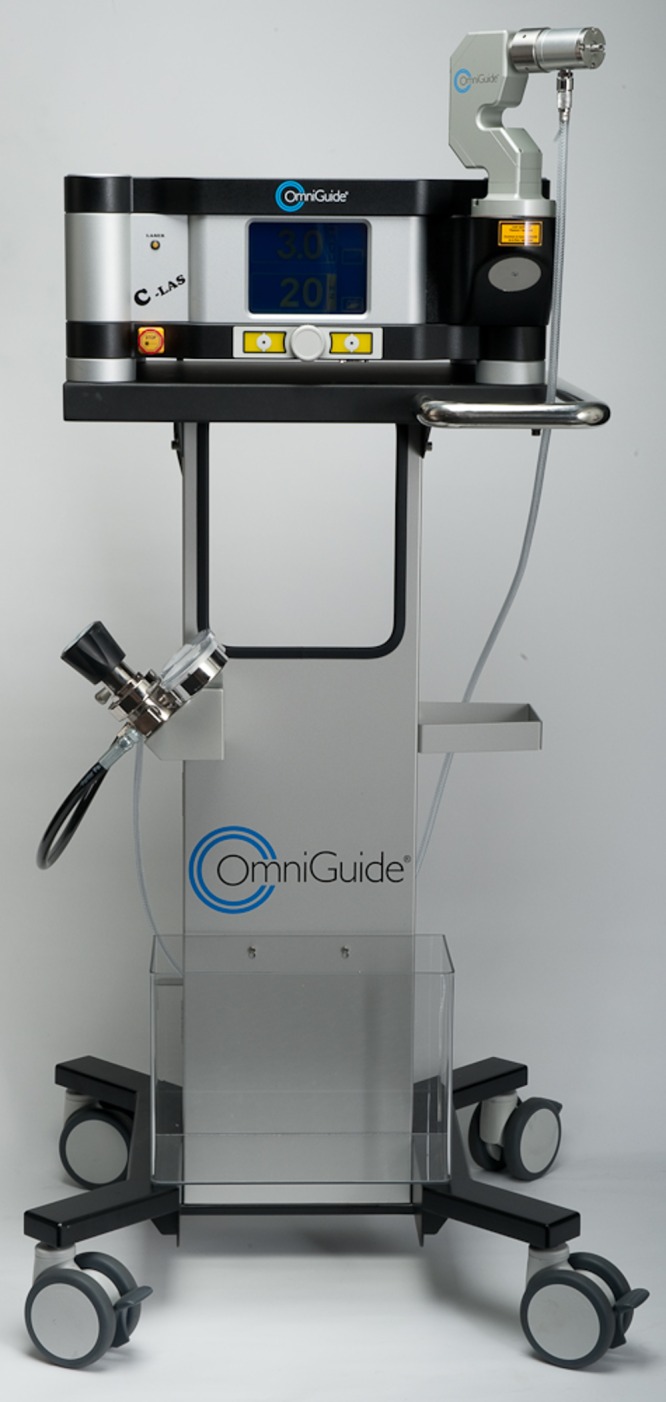
Power source of the CO2 laser (Omniguide, Boston, MA)

**Figure 3:**
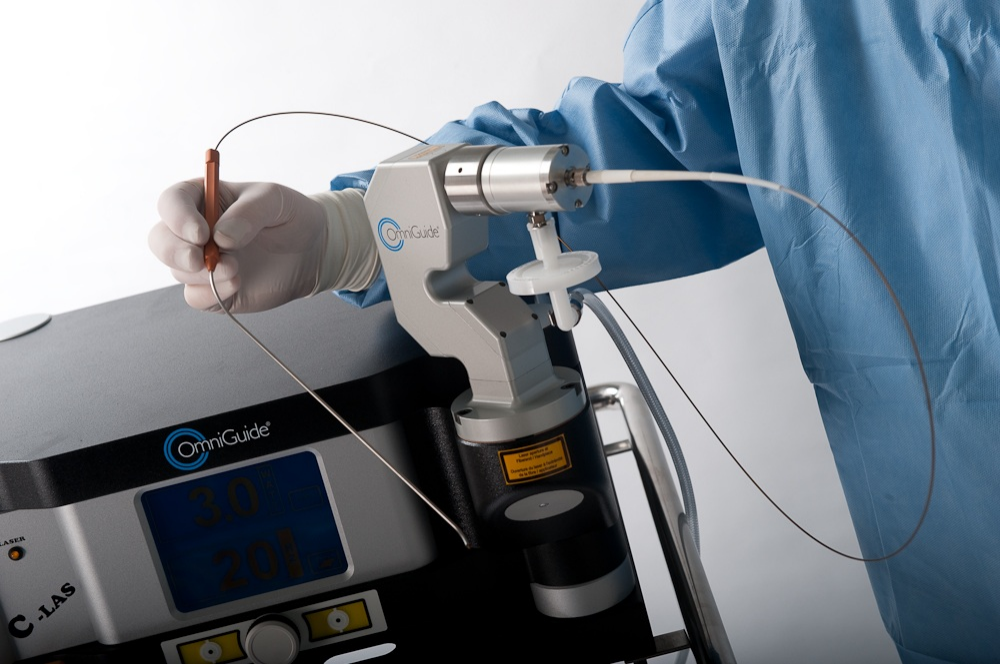
Handheld delivery with flexible fiber-optic wire connection to laser source

The contralateral ligament serves as a buffer and protects the dura when drilling across to the contralateral facet complex. After adequate bony decompression, attention can be turned to the ligamentum flavum on the contralateral side. Excessive retraction on the ligament can lead to dural tears, so care must be taken to desiccate and shrink the ligament to provide more working space down the tubular dilator. The CO_2_ laser can be used at this point to desiccate the ligament very effectively all the way to its connections at the far lateral facet and its junction to both the lamina and around the lateral extent of the bony canal. The laser console is shown in Figure 2, and the fiber-optic probe in Figure 3.

This laser functions well to stop any bone bleeding that may have begun after the bony decompression. After the hypertrophied bulky ligamentum flavum has been desiccated in this fashion, there is more working space behind the dura. The ligament itself has improved handling characteristics, being a little stiffer so as to facilitate biting portions of it and removing it in a piecemeal fashion with sharp biting instruments such as the Kerrison rongeur and sharp curettes.

Again, use of the CO_2_ laser does not make dural injury impossible. Reckless removal of the ligament and a lack of knowledge of the proper surgical anatomy and of this patient’s pathologic anatomy in particular as seen on preoperative imaging can pose unacceptable risks to any patient. All of the images should be reviewed, and the surgeons must have the experience and correct 3-dimensional visualization of the pathology for proper removal of the compressing forces in a safe, effective fashion. There is definitely a learning curve to overcome with regard to minimally invasive spine surgery and working through tubular dilators. Surgeons should only attempt these surgeries after appropriate training to the point where they feel very comfortable not only in dealing with surgical pathology but also in dealing with common surgical complications that may arise in the region being treated.

The laser, just as other surgical instruments being utilized through small working channels near sensitive anatomic structures, should be used judiciously and with caution. Injury to nerve roots, dural tear, devitalization of the muscle, and directly burning nervous tissue are all risks with the laser. One must also remember that reflection of the laser onto other instruments and for example, heating up the sucker, can cause complications. Many heating complications can be treated with irrigation. In addition, other problems can be managed in traditional ways. Dural tears and spinal fluid leaks should be explored for the possibility of primarily closing the defect. Dural sealants can be used, along with lumbar drains.

Close study of the preoperative MRI to examine specific levels of and degrees of stenosis and the status of the ligamentum flavum will help with preoperative planning and to determine whether the laser would be helpful. Careful study of the preoperative and intraoperative images will also facilitate the approach and help to reduce complications. 

The patient is a 66-year-old male with a long, several-year history of low back pain coupled with several months of neurogenic claudication. He can walk only around 100 yards before he begins experiencing severe cramping and pain in the low back and posterior aspect of the thighs and legs. He has undergone multiple conservative measures, including rest, physical therapy, stretching, and epidural injections. Informed patient consent was obtained prior to treatment. No reference to the patient's identity is present in this paper.

**Figure 4:**
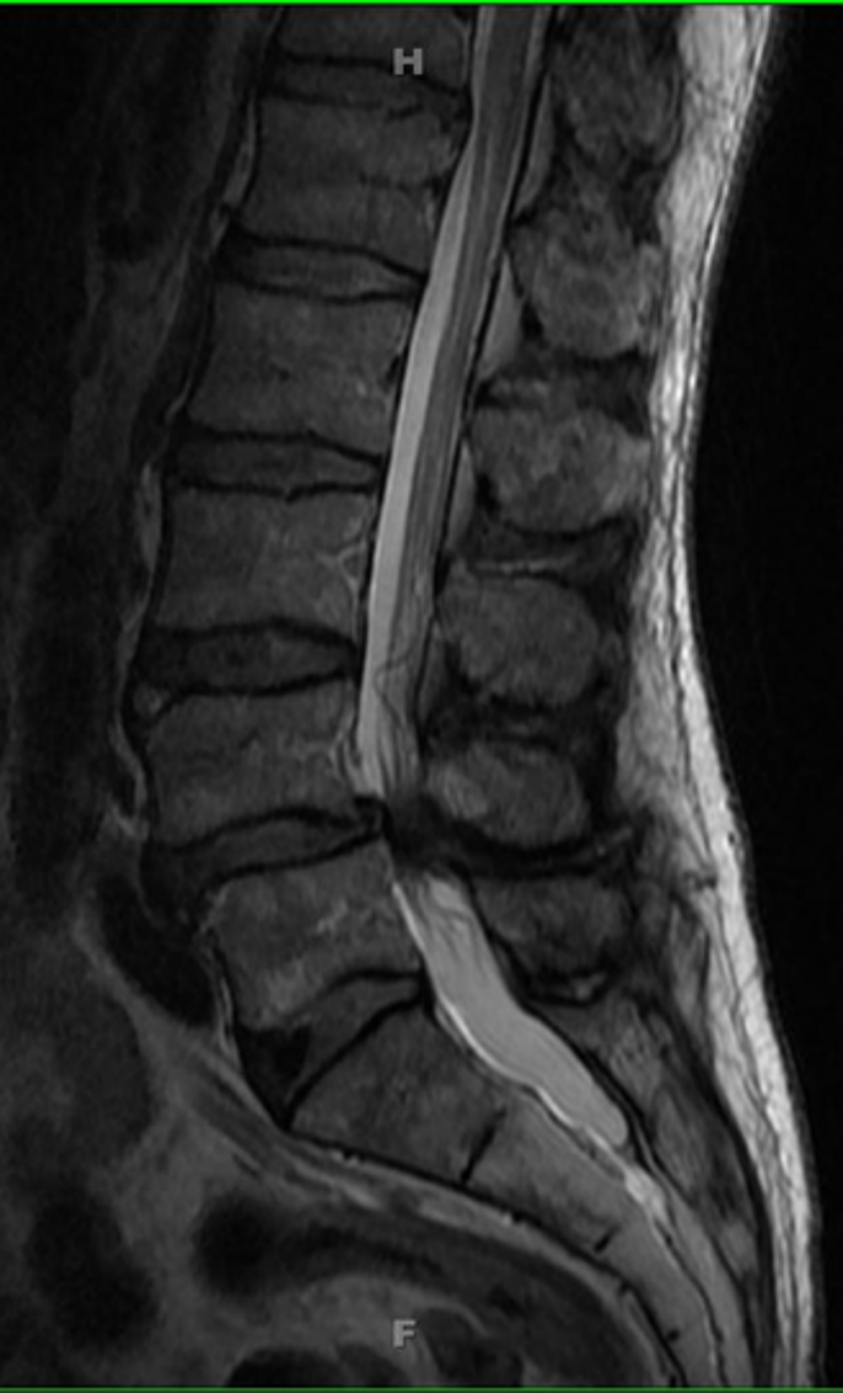
MRI, sagittal T2 MRI imaging reveals severe multilevel spinal stenosis with severely hypertrophied ligamentum flavum

**Figure 5:**
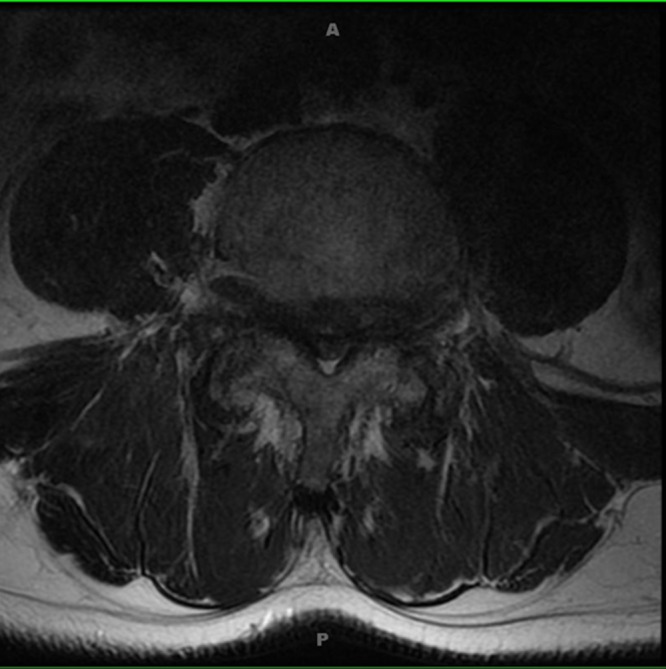
MRI, axial T2

MRI imaging as shown in Figure 4 (sagittal T2) and Figure 5 (axial T2) reveals severe spinal stenosis and spondylolisthesis with severely hypertrophied ligamentum flavum.

**Figure 6:**
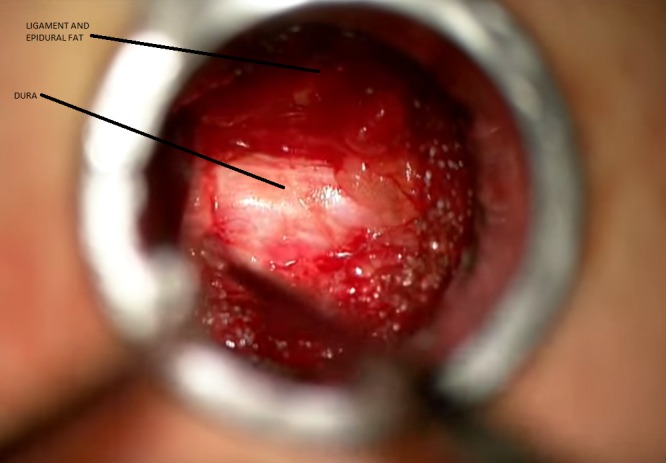
Dura visible after laser ablation of the contralateral ligamentum flavum

Nerve roots and the cauda equina appear even clumped due to the severe canal stenosis. The patient was taken to the operating room for a laminectomy, with good postoperative decompression as shown in Figure 6.

The patient’s symptoms resolved, and he is improving with respect to his exercise tolerance.

## Discussion

Several groups have reported good surgical results with the CO_2_ laser [2-4]. This technology has the potential to reduce complications and decrease the need to place delicate neural structures at risk [5]. The laser has been used in many applications, including otology, laryngology, head and neck surgical oncology, and spine surgery [6-8].

Temelkuran originally designed the hollow optical fiber lined with omnidirectional dielectric mirrors. Light is trapped within the hollow core by establishing large photonic bandgaps by alternating thin layers of high- and low-refractive index materials [9-10]. Omniguide fiber, which delivers the beam through flexible hollow-core photonic bandgap mirror fibers, allows the laser to be wielded like any other surgical instrument. This technological advance made the use of the laser in the operating room truly feasible. The first clinical surgical applications were in the field of otolaryngology [3, 11].

Waveform and energy dissipation characteristics are favorable with respect to tissue interactions and heat transfer. Ryan et al., have extensively studied the interactions between the laser and tissue, comparing it with bipolar-tissue interactions as well [12]. Water has a high absorption of energy from the CO_2_ laser, so a dry operative field is required. It cannot penetrate water or CSF, so intraventricular applications are limited.

In a recent report from Barrow Neurological Institute, investigators report a large series of 11 cavernous malformations, 14 meningiomas, seven ependymomas, three metastases, three astrocytomas, and seven other lesions where surgeons utilized the laser with success [4]. Surgeons rated the utility of the technology as 3.7 on a scale of 1 to 5. Browd, et al., found the laser to be useful with regard to spinal cord untethering procedures [2]. A point regarding the inability of the CO_2_ laser to travel through a cottonoid patty made such surgeries possible. Otherwise, dural damage would be risked.

Our case series provides further evidence of that safety of using the CO_2_ laser for yet another neurosurgical application: desiccating and removing the ligamentum flavum during minimally invasive spinal decompressive procedures. Out of our series of 40 consecutive levels being studied, we have not had one incident of dural tear, nerve root injury, muscle injury, new neurological deficit, or any other complication, which compares favorably to the 13% dural tear incidence reported in some studies [13]. This preliminary study provides evidence that the CO_2_ laser can be used as a useful adjunct during minimally invasive laminectomies for stenosis. Larger, randomized studies with multiple surgeons will be necessary to more definitely prove its benefits and justify its costs.

## Conclusions

The flexible CO_2_ laser is well suited for removal of the ligamentum flavum and surrounding soft tissues during decompressive procedures for lumbar stenosis, providing the appropriate exposure and decreasing risk of complications. 
